# The DNMT3A PWWP domain is essential for the normal DNA methylation landscape in mouse somatic cells and oocytes

**DOI:** 10.1371/journal.pgen.1009570

**Published:** 2021-05-28

**Authors:** Kanako Kibe, Kenjiro Shirane, Hiroaki Ohishi, Shuhei Uemura, Hidehiro Toh, Hiroyuki Sasaki

**Affiliations:** 1 Division of Epigenomics and Development, Medical Institute of Bioregulation, Kyushu University, Fukuoka, Japan; 2 Department of Stem Cell Biology and Medicine, Graduate School of Medical Sciences, Kyushu University, Fukuoka, Japan; 3 Graduate School of Integrated Science for Life, Hiroshima University, Higashi-Hiroshima, Japan; University of Pennsylvania, UNITED STATES

## Abstract

DNA methylation at CG sites is important for gene regulation and embryonic development. In mouse oocytes, *de novo* CG methylation requires preceding transcription-coupled histone mark H3K36me3 and is mediated by a DNA methyltransferase DNMT3A. DNMT3A has a PWWP domain, which recognizes H3K36me2/3, and heterozygous mutations in this domain, including D329A substitution, cause aberrant CG hypermethylation of regions marked by H3K27me3 in somatic cells, leading to a dwarfism phenotype. We herein demonstrate that D329A homozygous mice show greater CG hypermethylation and severer dwarfism. In oocytes, D329A substitution did not affect CG methylation of H3K36me2/3-marked regions, including maternally methylated imprinting control regions; rather, it caused aberrant hypermethylation in regions lacking H3K36me2/3, including H3K27me3-marked regions. Thus, the role of the PWWP domain in CG methylation seems similar in somatic cells and oocytes; however, there were cell-type-specific differences in affected regions. The major satellite repeat was also hypermethylated in mutant oocytes. Contrary to the CA hypomethylation in somatic cells, the mutation caused hypermethylation at CH sites, including CA sites. Surprisingly, oocytes expressing only the mutated protein could support embryonic and postnatal development. Our study reveals that the DNMT3A PWWP domain is important for suppressing aberrant CG hypermethylation in both somatic cells and oocytes but that D329A mutation has little impact on the developmental potential of oocytes.

## Author summary

Epigenetic modifications of histones and DNA play important roles in gene regulation and embryonic development. It is increasingly evident that interplay between the modifications is important for shaping their distribution patterns. We herein examine the role of the PWWP domain of DNMT3A, the major DNA methyltransferase expressed in mammalian oocytes, in shaping the oocyte-specific DNA methylation landscape. The PWWP domain is known to recognize di- and tri-methylated lysine 36 of histone H3 (H3K36me2/3), the latter of which is a histone modification especially important for shaping the DNA methylation landscape in oocytes. We found that, despite the potential importance of the PWWP domain in H3K36me3-dependent DNA methylation, mouse DNMT3A protein with an amino acid substitution disrupting the H3K36me3 recognition has little impact on the DNA methylation of H3K36me3-marked regions. Instead, the mutated protein causes aberrant DNA hypermethylation in regions lacking H3K36me2/3, including H3K27me3-marked regions. Furthermore, mouse oocytes that only express the mutated protein can support normal development when fertilized with wild-type sperm. Our findings suggest that DNMT3A has a backup system to recognize H3K36me3 without a normal PWWP domain and that mouse embryos have the potential to tolerate or reprogram the aberrant DNA hypermethylation caused by the PWWP mutation.

## Introduction

DNA methylation is an epigenetic modification that regulates the expression of genes by modulating chromatin conformation and transcription factor binding [1]. It is essential for normal mammalian development and its defects or misregulation can cause developmental abnormalities and cancer [2,3]. DNA methylation predominantly occurs at cytosines of CG dinucleotides, resulting in 5-methylcytosine (5mC); however, CH (H  =  A, T, or C) methylation also exists in specific cell types, including oocytes [4–10]. Several DNA methyltransferases are present in mice: DNMT1 is the maintenance methyltransferase that copies preexisting CG methylation patterns upon replication [11], while DNMT3A, DNMT3B and DNMT3C are *de novo* methyltransferases that create new methylation patterns [12–14]. DNMT3L lacks catalytic activity but enhances the activity of DNMT3A and DNMT3B through direct interaction [15–18].

Among mouse tissues and cells, oocytes have some unique features regarding the DNA methylation landscape. Previous studies by whole genome bisulfite sequencing (WGBS) revealed that fully grown oocytes (FGOs) have a significantly lower global CG methylation level in comparison to most other cell types (~40% vs. ~70%) and that a high level of methylation is confined to actively transcribed regions [19–21]. Interestingly, maternally methylated imprinting control regions also reside in the transcribed regions [21,22]. Such regions are marked by the trimethylation of histone H3 at lysine 36 (H3K36me3) [23] and depletion of this histone mark results in a global loss and redistribution of CG methylation in mouse oocytes [24]. Thus, CG methylation is clearly dependent on H3K36me3 in oocytes. In addition, FGOs are extremely rich in CH methylation: 75% of all 5mCs occur in a CH context [8,10]. Both CG methylation and CH methylation occur during oocyte growth [10,19,20] and are mediated by the DNMT3A-DNMT3L complex [10,15,16,20,25,26]. DNMT3A is expressed in two isoforms: DNMT3A1 is the full-length protein and DNMT3A2 is a predominant isoform in oocytes lacking an N-terminal portion [27,28]. Disruption of either *Dnmt3a* or *Dnmt3l* in mouse oocytes causes misregulation of maternally imprinted genes, leading to embryonic lethality [15,16,25,26]. Some other factors are also involved in the *de novo* methylation process; however, their contribution seems limited [10,29,30].

DNMT3A proteins have a Pro-Trp-Trp-Pro (PWWP) domain that is important for the recognition of H3K36me2/3 [31–37] and localization to the major satellite repeat at pericentromeres [38,39]. Recently, missense mutations were found within this domain in patients with microcephalic dwarfism [40]. The mutations (W330R and D333N) resulted in reduced binding to H3K36me2/3 and caused growth failure and CG hypermethylation of a subset of Polycomb-targeted regions, marked by H3K27me3, in heterozygous patients. The phenotype was recapitulated in a mouse model carrying a W326R substitution (corresponding to human W330R) [40]. An independent study showed that mice that were heterozygous for D329A mutation (at a position corresponding to human D333) exhibit postnatal growth retardation, CG hypermethylation in H3K27me3-marked and H3K4me3/H3K27me3 co-marked (bivalent) chromatin, and derepression of developmental genes in adult hypothalamus [41]. The study also suggested that heterozygous females have a parturition problem, resulting in a maternal transmission deficit. These results suggest that the major function of the DNMT3A PWWP domain is to limit CG methylation in H3K27me3-marked regions. Notably, in these mutants, H3K36me2/3-marked regions showed very limited loss of CG methylation.

Despite the potential importance of the DNMT3A PWWP domain in H3K36me-dependent *de novo* DNA methylation in oocytes [23,24], this has not been explored, perhaps due to the breeding difficulties of the mutant mice. We studied the effect of the D329A substitution in mouse oocytes with the combinatorial use of this mutation and oocyte-specific *Dnmt3a* depletion, and report that the PWWP domain is essential for suppressing aberrant DNA methylation in oocytes.

## Results

### Generation and the phenotypic analysis of *Dnmt3a*^*D329A*^ mice

**Fig 1:**
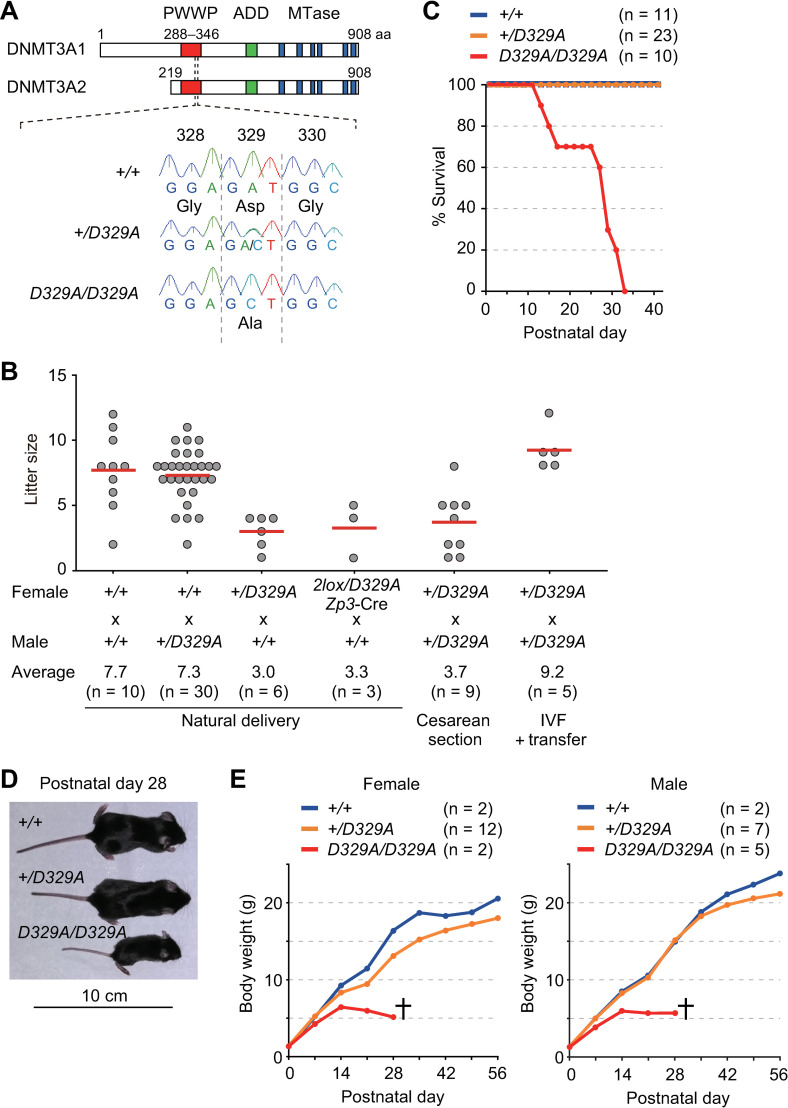
The generation and phenotypic analysis of *Dnmt3a*^*D329A*^ mice. (A) The structure of mouse DNMT3A (DNMT3A1 and DNMT3A2) and the position of D329A substitution. Known domains are indicated by colored boxes. Oocytes express both DNMT3A1 and DNMT3A2 while most other cell types only express DNMT3A1. An example of genotyping by Sanger sequencing is shown. (B) Litter size at birth obtained upon various crosses. Horizontal bars represent the mean litter size. (C) Postnatal survival of pups of each genotype. (D) Gross morphology of mice representative of each genotype. (E) Body weight changes of each genotype during postnatal development.

We generated mutant mice carrying an aspartic acid (GAT) to alanine (GCT) substitution at codon 329 (D329A) of the DNMT3A PWWP domain (*Dnmt3a*^*D329A*^ mice) [31,37,41] using CRISPR/Cas9-mediated homology directed repair (see Materials and Methods) (Fig 1A). Since the PWWP domain is present in common in both DNMT3A1 and DNMT3A2, this substitution affects both isoforms. Consistent with the previous report [41], *Dnmt3a*^*+/D329A*^ mice showed a dwarfism phenotype and such females crossed with wild-type males gave birth to fewer pups (mean litter size: 3.0) (Fig 1B). Furthermore, all pups derived from heterozygous females died before postnatal day 4 (P4). In contrast, heterozygous males were fully fertile.

Previous studies on *Dnmt3a*^*W326R*^ and *Dnmt3a*^*D329A*^ mice did not report homozygous mice [40,41], perhaps due to breeding difficulties. To obtain *Dnmt3a*^*D329A/D329A*^ mice, we intercrossed heterozygous mice and performed Caesarean section. Although the mean litter size was small (3.7) (Fig 1B), pups of all expected genotypes, including homozygotes, were obtained at a near Mendelian ratio (S1A Fig). Some of the pups were then fostered by lactating ICR females, and 30.3% (10/33) of them survived beyond weaning (P28). Thus, the early postnatal lethality of the pups delivered by heterozygous females was partially rescued by fostering. However, all seven homozygotes died before weaning.

We then performed *in vitro* fertilization (IVF) of *Dnmt3a*^*+/D329A*^ oocytes with *Dnmt3a*^*+/D329A*^ sperm and transferred resulting 2-cell embryos to the oviducts of pseudo-pregnant ICR females (S1B Fig). This fully recovered the litter size at birth (Fig 1B), with pups of all genotypes observed at a near Mendelian ratio (S1A Fig), indicating that the fetal loss was a maternal phenotype. We then traced the survival of the pups and found that all homozygotes died before P32, while all others survived beyond this stage (Fig 1C). The homozygotes showed postnatal growth retardation and were even smaller than heterozygotes (Fig 1D and 1E). These results indicate that, while inappropriate feeding or maternal care by the heterozygous mothers accounts for the early postnatal lethality, subsequent survival and degree of dwarf phenotype are dependent on the pup’s genotype.

### Aberrant CG hypermethylation in *Dnmt3a*^*D329A*^ somatic cells

**Fig 2:**
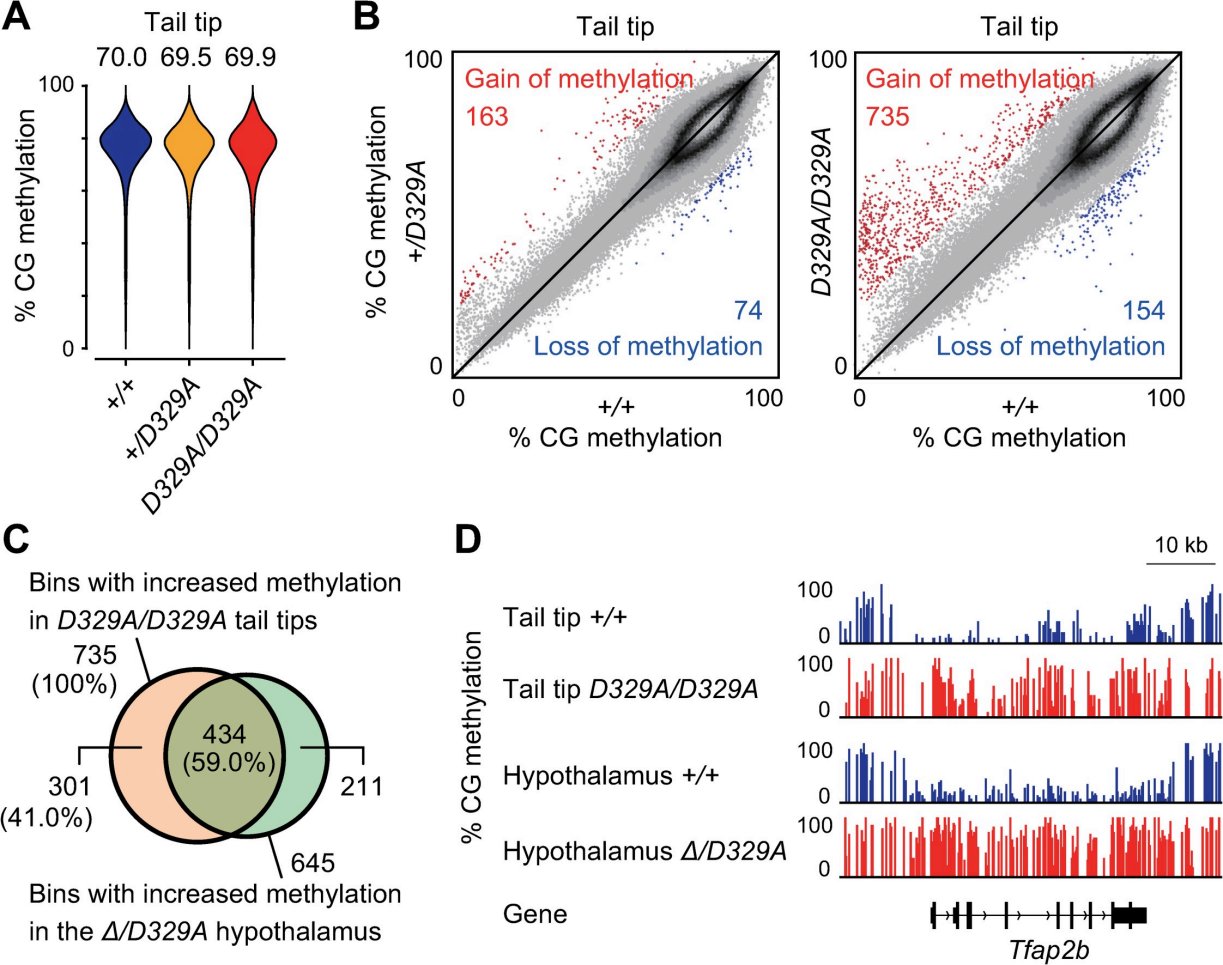
Aberrant CG hypermethylation in *Dnmt3a*^*D329A*^ tail tips. (A) Bean plots showing the distributions of CG methylation levels of 10-kb bins in the tail tips of the indicated genotypes. The number above each plot indicates the global CG methylation level. (B) Scatter plots comparing the CG methylation levels of 10-kb bins between the tail tips of indicated genotypes. Bins showing a ≥20% increase and those showing a ≥20% decrease in mutants in comparison to wild-type controls are shown by red and blue dots, respectively. (C) A Venn diagram showing the overlap between hypermethylated 10-kb bins of *Dnmt3a*^*D329A/D329A*^ tail tips and those of the *Dnmt3a*^*Δ/D329A*^ hypothalamus. (D) A genome browser view of a region showing aberrant CG hypermethylation in tail tips and the hypothalamus. Published data were used for the CG methylation profiles of hypothalamus [41].

Previous studies reported that *Dnmt3a*^*+/W326R*^ and *Dnmt3a*^*+/D329A*^ mice show aberrant CG hypermethylation in somatic cells [40,41]. While *Dnmt3a*^*Δ/D329A*^ mice also show CG hypermethylation [41], the methylation status is unknown in homozygous cells. We performed WGBS on tail tip DNA from wild-type, *Dnmt3a*^*+/D329A*^, and *Dnmt3a*^*D329A/D329A*^ pups obtained at P0 (S1 Table and S1B Fig). For all genotypes, we confirmed a good correlation in CG methylation distribution (non-overlapping 10-kilobase (kb) bins) between the replicate samples (S1 Table). While the three genotypes showed similar global levels and distribution patterns of CG methylation (Fig 2A), a small subset of bins showed >20% CG methylation changes in heterozygotes or homozygotes in comparison to wild-type mice (Fig 2B). In both genotypes, bins with higher methylation outnumbered those with lower methylation; however, homozygotes showed more higher methylation bins. Notably, 59.0% (434/735) of the bins showing increased methylation in the tail tips of homozygotes overlapped with those in the *Dnmt3a*^*Δ/D329A*^ hypothalamus [41] (Fig 2C and 2D).

### Generation of oocytes expressing only *Dnmt3a*^*D329A*^

Since homozygotes were extremely small and were lost by P32, we were not able to test their fertility. We therefore generated [*Dnmt3a*^*2lox/D329A*^, *Zp3*-Cre] mice, in which early growing oocytes undergo *Dnmt3a*^*2lox*^ to *Dnmt3a*^*1lox*^ conversion (via Cre-mediated, oocyte-specific deletion resulting in a frameshift) [26].

When [*Dnmt3a*^*2lox/D329A*^, *Zp3*-Cre] females were crossed with wild-type males, a reduced litter size comparable to that of heterozygous females was observed (Fig 1B). Furthermore, most pups (8/10) derived from such females died before P2, irrespective of the genotype. The two survivors (a *Dnmt3a*^*1lox/+*^ male and a [*Dnmt3a*^*D329A/+*^, *Zp3*-Cre] female) were among the five pups fostered by a lactating ICR female, again suggesting the need for appropriate feeding and care for pup survival. The results also suggest that *Dnmt3a*^*1lox/D329A*^ oocytes can support fetal development and postnatal survival under appropriate conditions. Importantly, efficient Cre-mediated disruption was confirmed by the absence of the *Dnmt3a*^*2lox*^ allele in all of the pups.

### Aberrant CG hypermethylation in *Dnmt3a*^*D329A*^ oocytes

**Fig 3:**
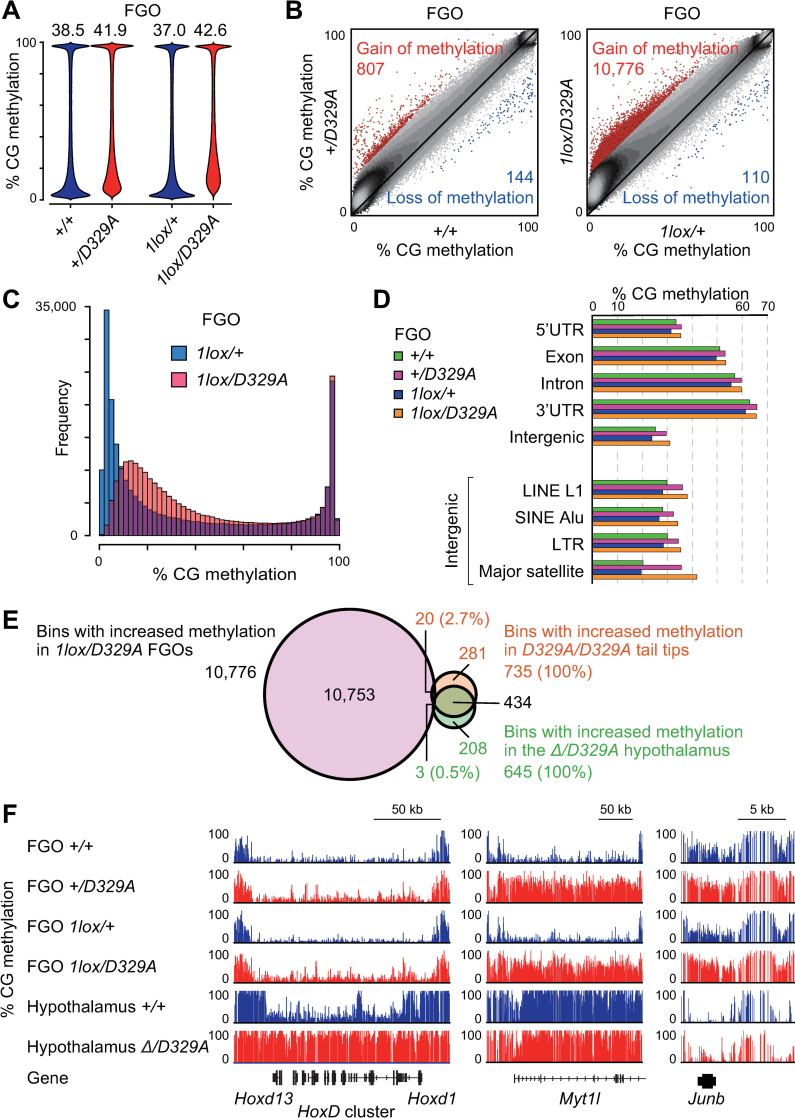
Aberrant CG hypermethylation in *Dnmt3a*^*D329A*^ oocytes. (A) Bean plots showing the distributions of CG methylation levels of 10-kb bins in FGOs of the indicated genotypes. The number above each plot indicates the global CG methylation level. (B) Scatter plots comparing the CG methylation levels of 10-kb bins between FGOs of the indicated genotypes. Bins showing a ≥20% increase and those showing a ≥20% decrease in mutants in comparison to controls are shown by red and blue dots, respectively. (C) Histograms showing the frequencies of 10-kb bins across CG methylation levels in *Dnmt3a*^*1lox/+*^ and *Dnmt3a*^*1lox/D329A*^ FGOs. (D) The CG methylation levels of indicated genomic annotations and repeats in FGOs of the indicated genotypes. Note that introns and intergenic regions contain various repeat sequences in addition to unique sequences. (E) A Venn diagram showing the overlaps between hypermethylated bins in indicated tissues of the indicated genotypes. (F) A genome browser view of regions showing CG hypermethylation (middle and right) and those showing little change (left) in mutant FGOs. Note that the *HoxD* cluster is hypermethylated in mutant hypothalamus but not in mutant FGOs.

To examine the impact of D329A substitution on the DNA methylation landscape of oocytes, we obtained FGOs from wild-type, *Dnmt3a*^*+/D329A*^, [*Dnmt3a*^*2lox/+*^, *Zp3*-Cre], and [*Dnmt3a*^*2lox/D329A*^, *Zp3*-Cre] females at 5–12 weeks and performed WGBS (S1 Table and S1B Fig). The genotypes of FGOs from [*Dnmt3a*^*2lox/+*^, *Zp3*-Cre] and [*Dnmt3a*^*2lox/D329A*^, *Zp3*-Cre] females were *Dnmt3a*^*1lox/+*^ and *Dnmt3a*^*1lox/D329A*^, respectively. We found that the global CG methylation level was lower in *Dnmt3a*^*1lox/+*^ FGOs (37.0%) in comparison to wild-type FGOs (38.5%) (Fig 3A), which is attributable to haploinsufficiency [41]. A similar CG methylation reduction was also observed in *Dnmt3a*^*1lox/+*^ FGOs from younger females (P25) (S2A Fig). It is known that wild-type FGOs show bimodal distribution of regional CG methylation levels [20,21]. The methylation loss in *Dnmt3a*^*1lox/+*^ FGOs was uniform across the genome, except for some 10-kb bins with extremely high or low CG methylation in wild-type FGOs (S3A Fig). Importantly, as observed in the hypothalamus and tail tip, the global CG methylation level was higher in *Dnmt3a*^*+/D329A*^ and *Dnmt3a*^*1lox/D329A*^ FGOs in comparison to their controls (wild-type and *Dnmt3a*^*1lox/+*^ FGOs, respectively) (Fig 3A) and we identified 807 and 10,776 bins with higher methylation in the respective genotypes (Fig 3B). Since 62.0% of the bins showing higher methylation in *Dnmt3a*^*+/D329A*^ FGOs were included in those identified in *Dnmt3a*^*1lox/D329A*^ FGOs (S3B Fig), we focused on aberrant hypermethylation in *Dnmt3a*^*1lox/D329A*^ FGOs (and *Dnmt3a*^*1lox/+*^ FGOs as control) in the subsequent analyses.

The gain of methylation in *Dnmt3a*^*1lox/D329A*^ FGOs primarily occurred at bins showing low to intermediate levels of methylation in control *Dnmt3a*^*1lox/+*^ FGOs (Fig 3B and 3C), which are termed hypomethylated or partially methylated domains [42]. This was also observed in FGOs from younger females (P25) (S2A and S2B Fig). These domains corresponded to intergenic or non-transcribed gene regions in oocytes [20,21]. In contrast, bins with high levels of methylation in control FGOs, corresponding to the actively transcribed regions [19–21], showed little change in *Dnmt3a*^*1lox/D329A*^ FGOs (Fig 3B and 3C). Among these regions were the maternally methylated imprinting control regions (S3C Fig). When various genomic annotations were individually examined, the major satellite repeat, corresponding to pericentromeric heterochromatin, showed the greatest gain of methylation in *Dnmt3a*^*1lox/D329A*^ FGOs (Fig 3D). This is consistent with the previous studies reporting that adequate DNA methylation of this repeat requires the DNMT3A PWWP domain [38,39]. Strikingly, only 2.7% (20/735) of the bins that showed higher methylation in the tail tips of homozygotes and only 0.5% (3/645) of the bins that showed higher methylation in the *Dnmt3a*^*Δ/D329A*^ hypothalamus [41] were more methylated in *Dnmt3a*^*1lox/D329A*^ FGOs (Fig 3E). Thus, the D329A mutation impacted different regions in somatic cells and oocytes. Examples are shown in Fig 3F: while the *Hox* gene cluster only gained methylation in somatic cells, the *Myt1l* and *Junb* genes only gained methylation in mutant FGOs.

### Limited impact of the D329A mutation on the transcriptome of oocytes

To examine whether the D329A mutation and observed CG hypermethylation have an impact on the transcriptome of FGO, we performed RNA sequencing (RNA-seq) with replicate FGO samples collected at 11–18 weeks (S2 Table). The transcriptomes were clustered according to the four genotypes (S4A Fig), and there was a good correlation between the replicate samples (S2 Table). When we sought for genes differentially expressed between the genotypes, only a small number of them was identified between wild-type and *Dnmt3a*^*+/D329A*^ FGOs and between *Dnmt3a*^*1lox/+*^ and *Dnmt3a*^*1lox/D329A*^ FGOs (false discovery rate <0.05, fold change ≥4) (S4B Fig and S3 Table). No significant enrichment for specific biological terms was observed with these genes. Thus, the D329A mutation had a limited impact on the transcriptome of FGOs. Importantly, there was no significant change in the levels of the other *Dnmt3* family members (S4C Fig) or the ratio of the *Dnmt3a* isoforms (S4D Fig), suggesting that the aberrant hypermethylation is likely due to the property of DNMT3A^D329A^ itself.

### Aberrant CG hypermethylation and histone H3 marks in oocytes

In human fibroblasts and the mouse hypothalamus, the DNMT3A PWWP mutations resulted in aberrant gain of CG methylation in chromatin regions marked by histone H3K27me3 or by both H3K27me3 and H3K4me3 (bivalent chromatin) [40,41]. It is known that FGOs have some unique features in their histone mark profile, such as the existence of distal H3K27me3 domains [42] and broad non-canonical H3K4me3 domains [43]. We therefore examined whether the aberrant hypermethylation is linked to any histone mark.

**Fig 4:**
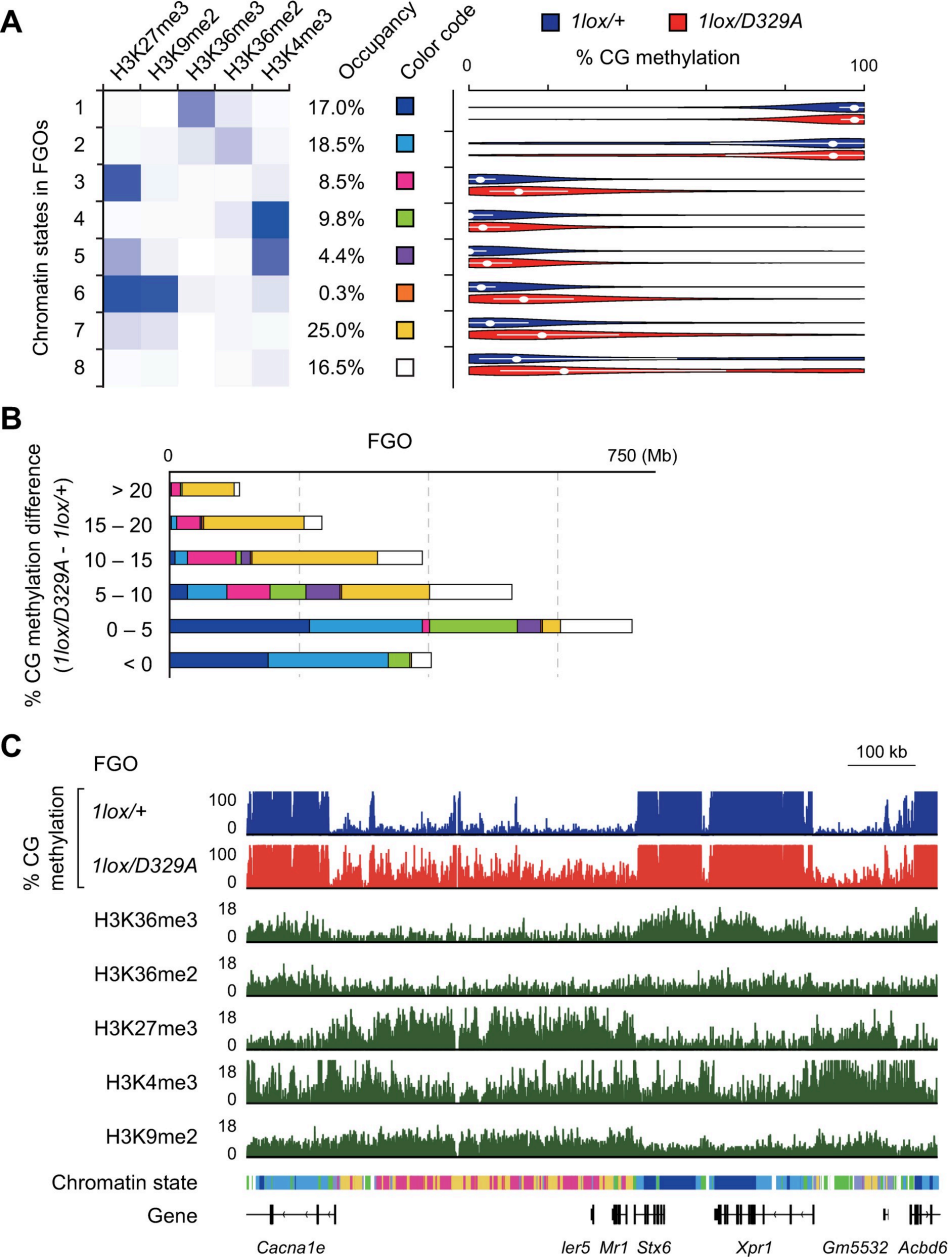
Aberrant CG hypermethylation and histone H3 marks in *Dnmt3a*^*D329A*^ oocytes. (A) Definition of an eight-state model based on five histone H3 marks in wild-type FGOs. Darker shades of blue in the emission profile represent greater enrichment of the histone mark in each chromatin state (left). The genomic occupancy of each chromatin state is also shown. Violin plots show the distribution of CG methylation levels (single CGs, sequencing depth >15) within regions labeled with each chromatin state in FGOs of the indicated genotypes (right). White dots and bars indicate the median and first and third quartiles, respectively. (B) Stacked bar charts showing the abundance of each chromatin state in the bulk of 10-kb bins with the indicated degrees of CG hypermethylation in *Dnmt3a*^*1lox/D329A*^ FGOs in comparison to *Dnmt3a*^*1lox/+*^ FGOs. The chromatin states are indicated by the color code shown in (A). (C) A genome browser view showing the CG methylation levels (%) in *Dnmt3a*^*1lox/D329A*^ FGOs and histone marks (RPM) and chromatin states in wild-type FGOs. The chromatin states are indicated below the histone mark profiles using the color code shown in (A).

A ChromHMM analysis [44] of publicly available chromatin immunoprecipitation sequencing (ChIP-seq) data for H3K36me3, H3K36me2, H3K27me3, H3K4me3, and H3K9me2 [30,45,46,47] revealed that the chromatin of FGO can be divided into eight states (Fig 4A). We then determined the regional CG methylation level of each state and found that H3K36me2/3-marked chromatin (states 1 and 2) is associated with a high level of methylation in both *Dnmt3a*^*1lox/+*^ and *Dnmt3a*^*1lox/D329A*^ FGOs (Fig 4A). This suggests that DNMT3A^D329A^ can recognize H3K36me2/3, directly or indirectly, and mediate *de novo* CG methylation. Among other chromatin states, those marked by H3K27me3 (state 3), co-marked by H3K27me3 and H3K9me2 (states 6 and 7), and lacking any strong mark (state 8) showed large increases in CG methylation in *Dnmt3a*^*1lox/D329A*^ FGOs (Fig 4A, 4B and 4C). The H3K4me3-marked state and bivalent state (states 4 and 5) showed only small increases in CG methylation (Fig 4A, 4B and 4C). Since it is known that H3K27me3-marked regions are normally resistant to CG methylation, we examined whether there is any change in expression of the Polycomb catalytic core components (*Eed*, *Ezh1*, *Ezh2*, and *Suz12*) or H3K27me3 demethylases (*Jarid2*, *Utx*, *Jmjd3*, and *Jhdm1d*). We found no such changes in our RNA-seq data (S4C Fig), indicating that the aberrant DNMT3A recruitment requires other explanations. Taken together, the PWWP domain of DNMT3A has an important role in limiting *de novo* CG methylation outside the H3K36me2/3-marked regions of oocytes. The bivalent domains of the oocytes were more resistant to aberrant hypermethylation (state 5) in comparison to those of the hypothalamus (state 4 of S5 Fig) [41].

### Aberrant CH hypermethylation in *Dnmt3a*^*D329A*^ oocytes

**Fig 5:**
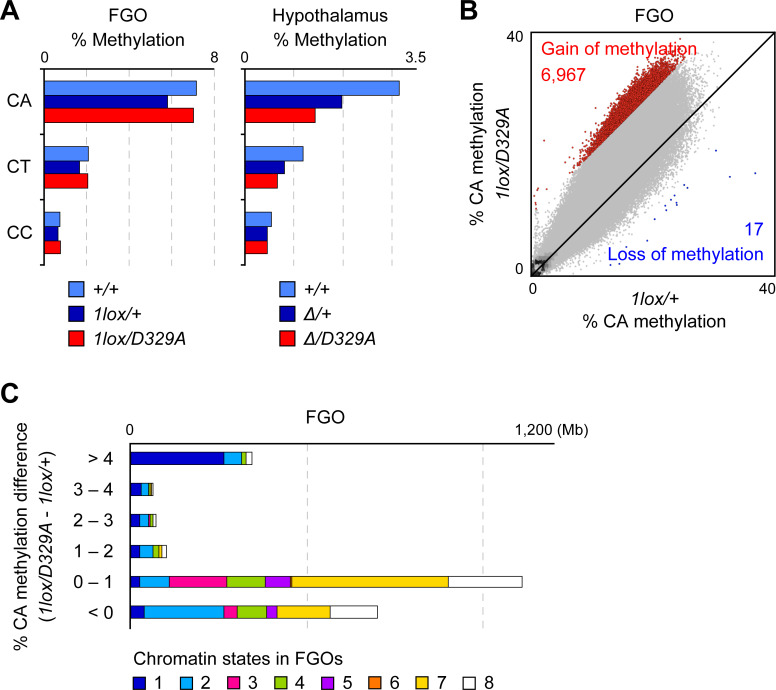
Aberrant CH methylation in *Dnmt3a*^*D329A*^ oocytes. (A) Bar graphs showing the methylation levels of the respective CH dinucleotides in FGOs (left) and the hypothalamus (right) of indicated genotypes. (B) Scatter plot comparing the CA methylation levels of 10-kb bins between FGOs of the indicated genotypes. Bins showing a ≥10% increase and those showing a ≥10% decrease in mutant FGOs in comparison to control FGOs are shown by red and blue dots, respectively. (C) Stacked bar charts showing the abundance of each chromatin state in the bulk of 10-kb bins with different degrees of CA hypermethylation in *Dnmt3a*^*1lox/D329A*^ FGOs in comparison to *Dnmt3a*^*1lox/+*^ FGOs. The chromatin states correspond to those defined in Fig 4A.

We previously reported that CH methylation accumulates in FGOs [8,10]. To study the impact of DNMT3A^D329A^ on CH methylation, we analyzed the WGBS data from wild-type, *Dnmt3a*^*1lox/+*^, and *Dnmt3a*^*1lox/D329A*^ FGOs. In all genotypes, CA sites were the most highly methylated among the CH sites (Fig 5A). The global CA methylation level was higher in *Dnmt3a*^*1lox/D329A*^ FGOs (7.2%) in comparison to *Dnmt3a*^*1lox/+*^ FGOs (5.9%), which was the opposite of the global CA hypomethylation reported in the *Dnmt3a*^*Δ/D329A*^ hypothalamus (Fig 5A) [41] (CH methylation was extremely low in tail tip DNA, close to the bisulfite conversion error rate, and we did not observe any difference between the genotypes). Although the CA methylation level of *Dnmt3a*^*1lox/D329A*^ FGOs was comparable to that of wild-type FGOs, which have two copies of wild-type *Dnmt3a*, the results suggested that DNMT3A^D329A^ has higher CA methylation activity in comparison to wild-type DNMT3A. The gain of CA methylation in *Dnmt3a*^*1lox/D329A*^ FGOs in comparison to *Dnmt3a*^*1lox/+*^ FGOs was remarkable in regions that showed high CA methylation and H3K36me3 enrichment in wild-type FGOs (Fig 5B and 5C). In contrast, such regions lost CA methylation in the *Dnmt3a*^*Δ/D329A*^ hypothalamus (S6A Fig). Lastly, our RNA-seq and next nucleotide analysis supported that the CH hypermethylation is indeed due to DNMT3A^D329A^, as there was no change in the expression of the other *Dnmt3* family members (S4C Fig) or in the next nucleotide preference (for example, if DNMT3B were to act as a major contributor, the preferred nucleotide after CH would be G, instead of C) [48] (S6B Fig).

## Discussion

The PWWP domain of DNMT3A recognizes histone H3K36me2/3 [31–37] and may be particularly important for *de novo* DNA methylation in oocytes, which primarily occurs at H3K36me3-marked regions [24]. It was previously reported that substitutions of an aspartic acid in the DNMT3A PWWP domain (D329A in mouse and D333N in human) results in postnatal growth retardation [40,41]. In this study, we generated *Dnmt3a*^*D329A*^ mice and advanced our knowledge on this domain by characterizing homozygous mutant mice and oocytes that only express DNMT3A^D329A^.

We first confirmed the dwarfism phenotype of *Dnmt3a*^*+/D329A*^ mice and the perinatal loss of offspring from heterozygous females. The latter phenotype was previously attributed to a parturition problem [41]; however, our study revealed a significant loss of fetuses during pregnancy. The prenatal loss was a maternal phenotype, since the litter size was fully recovered upon IVF and embryo transfer. Heterozygous females also had a problem in maternal behavior, but we did not explore this phenotype further. Importantly, IVF followed by embryo transfer enabled us to recover live homozygotes, which has not previously been reported. The homozygotes were even smaller than the heterozygotes and all of them died before P32. Furthermore, WGBS of the tail tip DNA revealed that homozygotes have intergenic CG hypermethylation that is more profound than that of heterozygotes. The regions hypermethylated in the tail skin of homozygotes largely (59.0%) overlapped with those that were hypermethylated in the *Dnmt3a*^*Δ/D329A*^ hypothalamus [41].

Since we were not able to obtain oocytes from homozygous females due to the early lethality phenotype, we generated [*Dnmt3a*^*2lox/D329A*^, *Zp3*-Cre] females, the oocytes of which only express DNMT3A^D329A^. Despite the fact that *de novo* CG methylation predominantly occurs in H3K36me3-marked regions [23,24] and that H3K36me2/3 recognition is severely affected by the D329A substitution [31,37], there was little, if any, loss of CG methylation in *Dnmt3a*^*1lox/D329A*^ oocytes. Notably, the maternally methylated imprinting control regions, which are transcribed and marked with H3K36me3 [21–24], remained highly methylated in *Dnmt3a*^*1lox/D329A*^ oocytes.

In contrast, the D329A substitution caused aberrant CG hypermethylation in regions lacking H3K36me2/3, including those marked by H3K27me3, which is mediated by the Polycomb repressive complex. These regions overlapped with the large hypomethylated regions previously described in oocytes [21]. We observed differences in response to the mutation between somatic cells and oocytes. Although bivalent domains co-marked with H3K27me3 and H3K4me3 are aberrantly CG hypermethylated in mutant somatic cells [40,41], this phenotype was weaker in mutant oocytes. The difference suggests that DNMT3A is less compatible with H3K4me3 in oocytes, which may be attributable to the predominant isoform expressed in each cell type (DNMT3A1 or DNMT3A2) or to the presence of oocyte-specific cofactor DNMT3L. While both DNMT3A and DNMT3L only interact with unmethylated H3K4 [49,50], the exact degree of incompatibility with H3K4me3 may differ between the isoforms and/or proteins. Thus, the DNMT3A PWWP domain protects regions lacking H3K36me2/3 from aberrant CG methylation in both somatic cells and oocytes, but there was almost no overlap between the affected regions. The lack of overlap is attributed to both cell type-specific histone modification patterns and DNMT3A’s cell type-specific responses to the modifications. We also found that among the various genomic annotations and repeats, the major satellite, potentially marked by H3K27me3 in FGOs [51], is also aberrantly CG hypermethylated in mutant oocytes. Lastly, while D329A substitution results in a loss of CH methylation in somatic cells [39], it caused a gain in CH methylation in oocytes. This may also be attributable to the cell-type-specific DNMT3A isoform or the presence of DNMT3L. Despite all these aberrations in DNA methylation, oocytes expressing only the mutated DNMT3A protein could support embryonic and postnatal development. A possible explanation is that the genome-wide reprogramming in cleavage stage embryos effectively erases the aberrant DNA hypermethylation; however, this requires further investigation.

Taken together with the findings from the previous studies [40,41], our results suggest that DNMT3A with a D329A substitution can somehow recognize H3K36me2/3 in somatic cells and oocytes, despite the greatly reduced interaction with H3K36me2/3 peptides *in vitro* [31,37]. Then, how does DNMT3A^D329A^ recognize H3K36me2/3? A trivial explanation is that the PWWP domain with D329A substitution has residual activity for interaction with H3K36me2/3, which is sufficient to introduce *de novo* CG and CH methylation in H3K36me2/3-marked regions. Another possibility is that DNMT3A indirectly recognizes H3K36me2/3 via, for example, DNMT3B (as it has a PWWP domain), which is expressed in oocytes and can form a complex with DNMT3A [28,52,53,54]. It could therefore help DNMT3A to target H3K36me2/3-marked regions.

In summary, our study reveals that the DNMT3A PWWP domain is important for the normal DNA methylation landscape of mouse oocytes but that D329A substitution has little impact on their developmental potential. The findings of the present study will provide further insight into how the DNA methylation landscape is established in mammalian oocytes.

## Materials and methods

### Ethics statement

Mouse husbandry and experiments were carried out in accordance with the ethical guidelines of Kyushu University and the protocols were approved by the Institutional Animal Care and Use Committee of Kyushu University. Mice subjected to molecular studies were euthanized by cervical dislocation.

### Generation of *Dnmt3a*^*D329A*^ mice

*Dnmt3a*^*D329A*^ mice were generated using a CRISPR/Cas9 method reported by Inui et al. [55]. Pronuclei of fertilized eggs obtained by crossing (C57Bl/6J x C3H) F1 females and males were injected with a pX330 plasmid (Addgene) encoding Cas9 and guide RNA and a single-stranded donor oligonucleotide. The oligonucleotide sequences used are shown in S4 Table. The injected zygotes were transferred to the oviducts of pseudo-pregnant ICR females (purchased from Kyudo). Genotyping of the pups by PCR-based Sanger sequencing of tail-tip DNA identified a male carrying an expected D329A substitution. This male was crossed with C57Bl/6J females to confirm successful germline transmission, and the offspring carrying the mutation (*Dnmt3a*^*+/D329A*^) was further backcrossed to C57Bl/6J mice. To obtain oocytes expressing only DNMT3A^D329A^ (with no wild-type DNMT3A), we generated [*Dnmt3a*^*2lox/D329A*^, *Zp3*-Cre] females and knocked out the *Dnmt3a*^*2lox*^ allele in an oocyte-specific manner [26]. *Dnmt3a*^*2lox*^ mice and *Zp3*-Cre mice were previously described [25,56]. Genotyping was performed by standard PCR or Eprobe (DNAFORM)-mediated real-time PCR monitoring coupled with a melting curve analysis. The primers used for genotyping are listed in S4 Table.

### IVF, embryo transfer, and collection of oocyte and tissue samples

Female mice (age: over 8 weeks) were injected with pregnant mare serum gonadotropin (7.5 IU) and then with human chorionic gonadotropin (7.5 IU) to induce superovulation. Cumulus-oocyte complexes were collected from the oviducts and IVF was performed according to the standard protocol. Fertilized eggs were incubated in a KSOM medium (Merck Millipore) at 37°C under 6% CO_2_. Two-cell embryos were transferred to the oviducts of pseudo-pregnant ICR females. Tail tips were obtained from the pups at P0 to monitor survival and body weight. FGOs were obtained from females at P25 and at over 5 weeks of age.

### WGBS and the data analysis

WGBS libraries were prepared using the post-bisulfite adapter tagging (PBAT) method [57]. Five hundred to one thousand FGOs and 200 ng of DNA from tail tips were respectively spiked with 0.03 ng and 2 ng of lambda phage DNA (Promega) and subjected to bisulfite conversion. The concentrations of the PBAT libraries were measured by qPCR using a KAPA Illumina Library Quantification kit (Kapa Biosystems). Cluster generation and sequencing were performed using a TruSeq SR Cluster kit v3-cBot-HS (Illumina) and a TruSeq SBS kit v3-HS (Illumina), according to the manufacturer’s protocols. The libraries were sequenced on a HiSeq 1500 equipped with HCS v2.2.68 and RTA v1.18.64 to generate 108-nucleotide single-end reads [58]. The adapter sequences and low-quality bases were removed from the 5’ and 3’ ends, respectively. The resulting reads were aligned to the reference mouse genome (mm10) using Bismark v0.10.0 [59]. The seed length was 28, the maximum number of mismatches permitted in the seed was 1, and the “—pbat” option was used. Only uniquely aligned reads were used for the subsequent analyses. Data from both strands were combined. We estimated the bisulfite conversion rate using reads that were uniquely aligned to the lambda phage genome. Sequences and information of chromosomes, RefSeq genes, CGIs, and repetitive elements of mouse (mm10) were downloaded from the UCSC genome browser. Bean plots and violin plots were generated to visualize the distribution of CG methylation levels using the R package v3.5.1 [60]. Raw fastq files of published WGBS datasets from the *Dnmt3a*^*+/+*^ and *Dnmt3a*^*Δ/D329A*^ hypothalamus (GSE117728) were downloaded from the Gene Expression Omnibus and were processed using our own pipeline.

### RNA-seq and the data analysis

Total RNA was obtained from a pool of 7–10 FGOs per each replicate. RNA-seq libraries were prepared using the SMART-Seq Stranded Kit (Takara Bio) according to the standard protocol [61,62]. In brief, total RNA was fragmented at 85°C for 6 min and then processed under the ultra-low-input workflow. PCR1 and PCR2 were respectively performed for 10 cycles, and final cleanup was performed twice. The libraries were sequenced on an Illumina NovaSeq 6000 using a NovaSeq 6000 SP Reagent Kit (paired-end 151 nt). Reads were trimmed and mapped to the reference mouse genome (mm10) by HISAT2 v2.1.0 [63]. Transcripts were assembled by StringTie v2.1.3 [64]. For hierarchical clustering and identification of the differentially expressed genes, iDEP online tools were used [65]. Read counts were filtered out by the criteria of at least 0.5 counts per million in one of the samples. The top 1,000 most variable genes were selected for clustering in the “Heatmap” module.

### The analysis of published ChIP-seq datasets

Raw fastq files of published ChIP-seq datasets for H3K36me3, H3K36me2, H3K27me3, H3K4me3, and H3K9me2 in wild-type FGOs (GSE93941, GSE148150, GSE112320, and GSE112622) and H3K36me3, H3K27me3, and H3K4me3 in the wild-type hypothalamus (GSE117728) were downloaded from the Gene Expression Omnibus. The raw sequence reads were trimmed to remove adapter sequences and low-quality bases and mapped to the reference mouse genome (mm10) using Bowtie v1.2.2 [66]. Chromatin seven-state models were generated by ChromHMM v1.18 [44] using the ChIP-seq data.

## Supporting information

S1 Fig Experimental design to investigate the role of the DNMT3A PWWP domain in mouse oocytes. (A) Genotypes of newborn pups obtained by intercrossing heterozygotes. The numbers in the graph indicate the numbers of pups obtained. (B) A schematic representation of the experimental design, including mouse crosses and analyses.
(PDF)

S2 Fig Aberrant CG hypermethylation in *Dnmt3a*^*D329A*^ oocytes collected at P25. (A) Bean plots showing the distributions of CG methylation levels of 10-kb bins in P25 FGOs of the indicated genotypes. The number above each plot indicates the global CG methylation level. (B) Scatter plots comparing the CG methylation levels of 10-kb bins between P25 FGOs of the indicated genotypes.
(PDF)

S3 Fig Aberrant CG methylation in *Dnmt3a*^*D329A*^ oocytes in comparison to other samples. (A) Scatter plots comparing the CG methylation levels of 10-kb bins between wild-type and *Dnmt3a*^*1lox/+*^ FGOs. Bins showing a ≥20% increase and those showing a ≥20% decrease in mutants in comparison to controls are shown by red and blue dots, respectively. (B) A Venn diagram showing the overlap between the hypermethylated bins in *Dnmt3a*^*+/D329A*^ and *Dnmt3a*^*1lox/D329A*^ FGOs. (C) The CG methylation levels of imprinting control regions in FGOs of the indicated genotypes.
(PDF)

S4 Fig Limited impact of the D329A mutation on the transcriptome of oocytes. (A) Cluster analysis of the transcriptomes from replicate FGO samples of the indicated genotypes. (B) Volcano plots showing genes differentially expressed between wild-type and *Dnmt3a*^*+/D329A*^ FGOs and those between *Dnmt3a*^*1lox/+*^ and *Dnmt3a*^*1lox/D329A*^ FGOs. The read counts are the average of the replicates. Blue dots represent down-regulated genes, whereas red dots represent up-regulated genes. (C) Expression of the Dnmt3 family members, Polycomb catalytic core components, and H3K27me3 demethylases in FGOs of the indicated genotypes. The transcript per kilobase million (TPM) values are the average of the replicates. (D) Expression of the Dnmt3a isoforms in FGOs of the indicated genotypes.
(PDF)

S5 Fig Aberrant CG hypermethylation and histone H3 marks in the *Dnmt3a*^*D329A*^ hypothalamus. Definition of a five-state model based on three histone H3 marks in the hypothalamus (top). Darker shades of blue in the emission profile represent greater enrichment of the histone marks in each chromatin state. The genomic occupancy of each chromatin state is also shown. Stacked bar charts showing the abundance of each chromatin state in the bulk of 10-kb bins with indicated degrees of CG hypermethylation in the *Dnmt3a*^*Δ/D329A*^ hypothalamus in comparison to the wild-type hypothalamus (middle). The bottom panel is a zoom-in view of the middle panel. The chromatin states are indicated by the color code shown at the top. Published histone H3 mark data of the wild-type hypothalamus were used to define the chromatin states [41].
(PDF)

S6 Fig Aberrant CA methylation in *Dnmt3a*^*D329A*^ oocytes and the *Dnmt3a*^*D329A*^ hypothalamus. (A) A genome browser view showing CA hypermethylation in *Dnmt3a*^*D329A*^ oocytes and CA hypomethylation in the *Dnmt3a*^*D329A*^ hypothalamus. CG methylation patterns are also shown. (B) Bar graphs showing the methylation levels of the respective CHH trinucleotides in FGOs (left) and the hypothalamus (right) of indicated genotypes.
(PDF)

S1 Table WGBS and mapping summary. (PDF)

S2 Table RNA-seq and mapping summary. (PDF)

S3 Table List of differentially expressed genes. (PDF)

S4 Table Oligonucleotide sequences. (PDF)
