# Supplementary figures and images for: The DNMT3A PWWP domain is essential for the normal DNA methylation landscape in mouse somatic cells and oocytes
Source: PLoS Genet. 2021 May 28;17(5):e1009570. doi: 10.1371/journal.pgen.1009570 (PMC8162659; doi:10.1371/journal.pgen.1009570)

**A**  $+/D329A$  ♀ ×  $+/D329A$  ♂ → Genotype of offspring

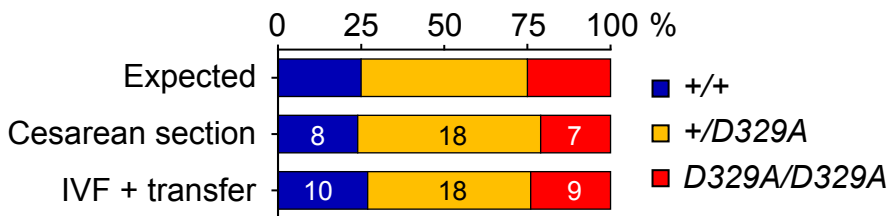

**B**

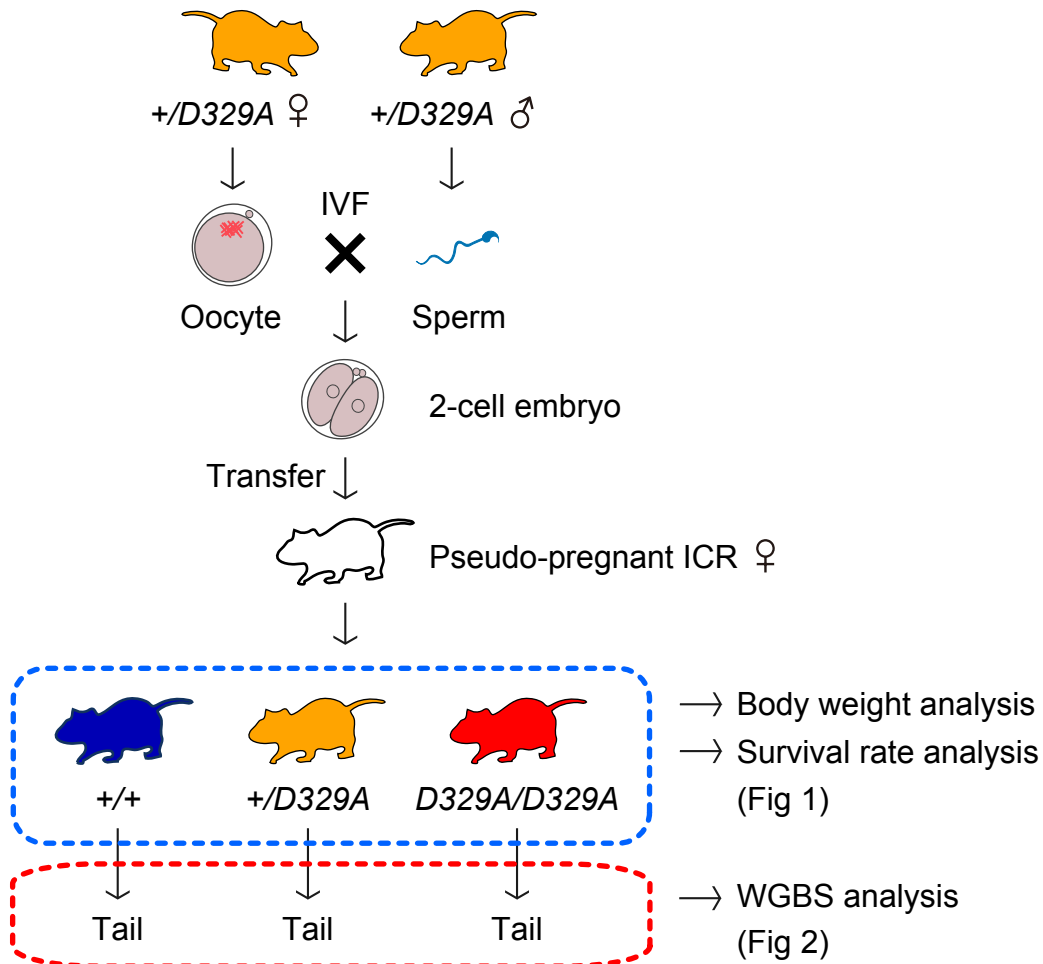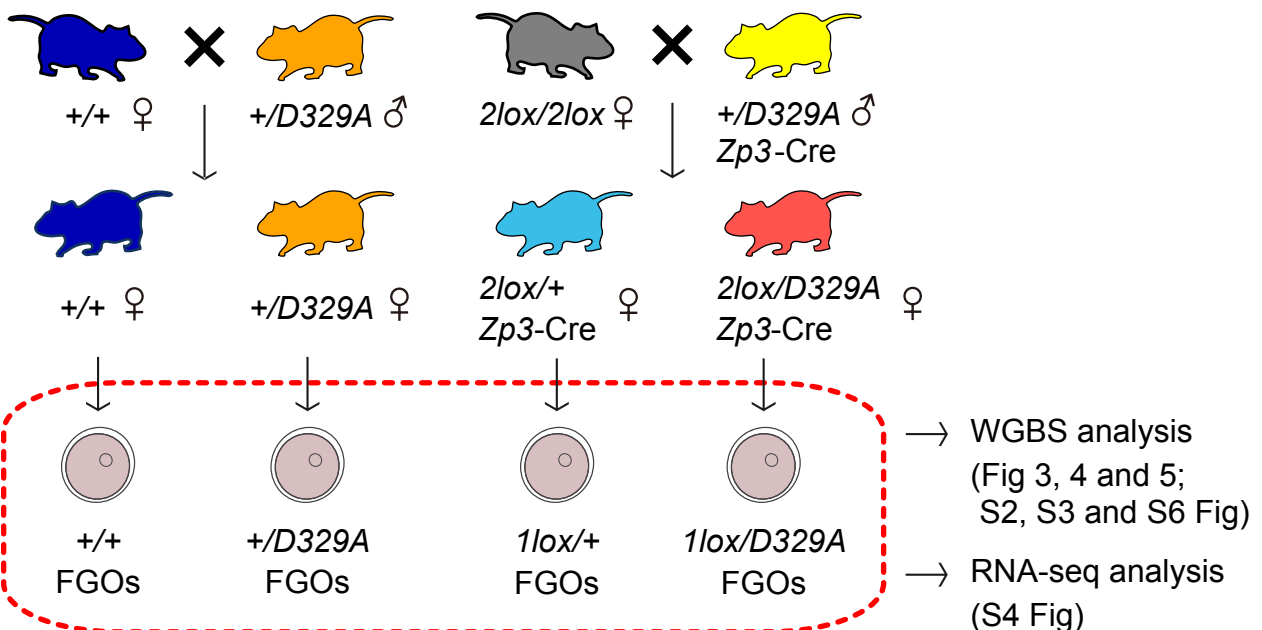

Supplement: S1 Fig — (A) Genotypes of newborn pups obtained by intercrossing heterozygotes. The numbers in the graph indicate the numbers of pups obtained. (B) A schematic representation of the experimental design, including mouse crosses and analyses. (PDF) [file pgen.1009570.s001.pdf]

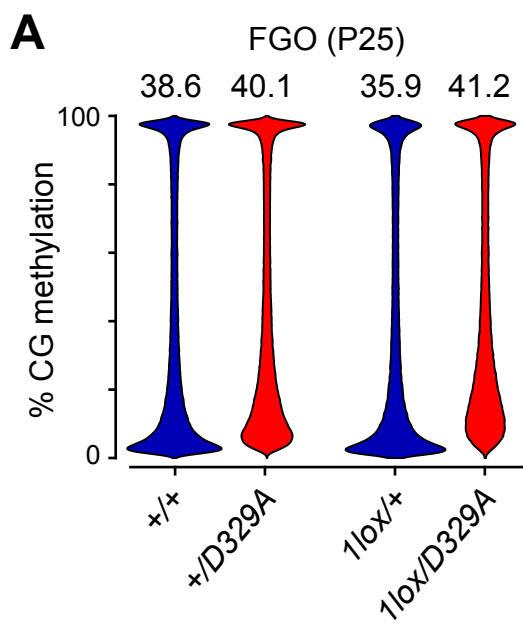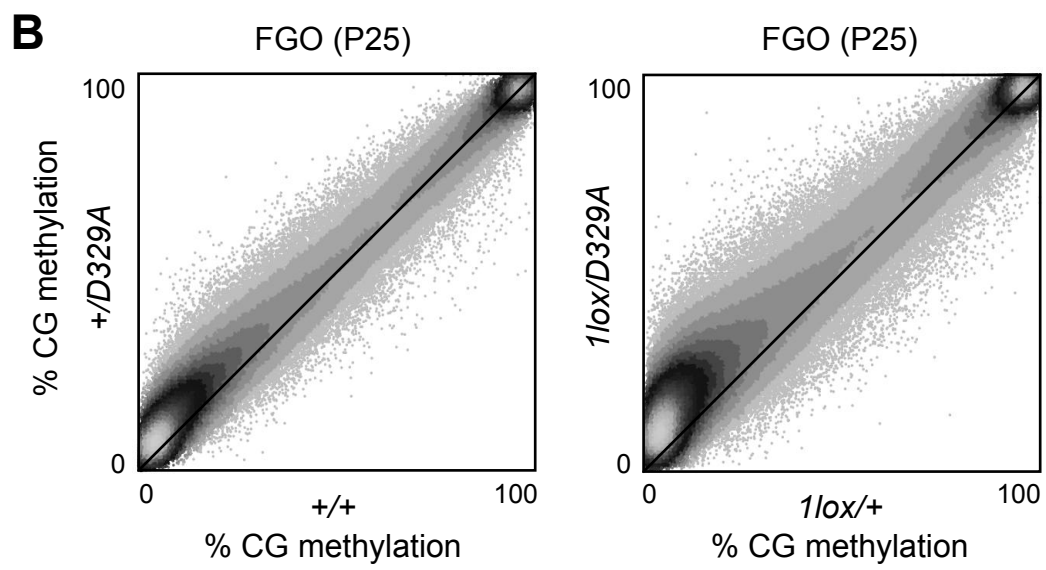

**S2 Fig**

Supplement: S2 Fig — (A) Bean plots showing the distributions of CG methylation levels of 10-kb bins in P25 FGOs of the indicated genotypes. The number above each plot indicates the global CG methylation level. (B) Scatter plots comparing the CG methylation levels of 10-kb bins between P25 FGOs of the indicated genotypes. (PDF) [file pgen.1009570.s002.pdf]

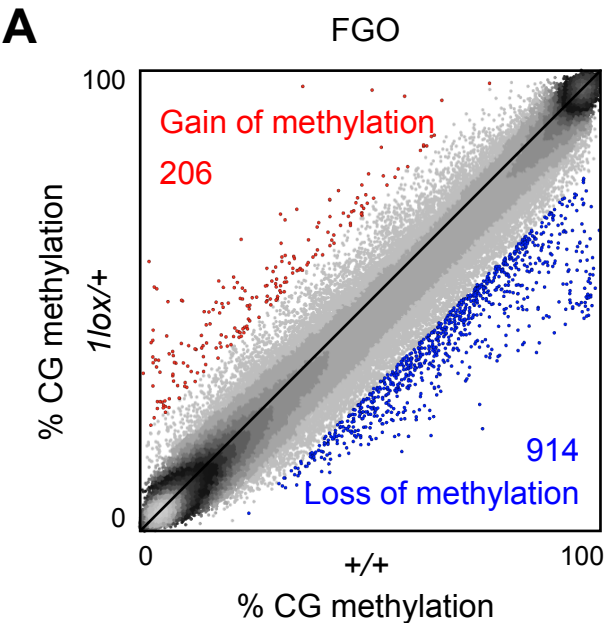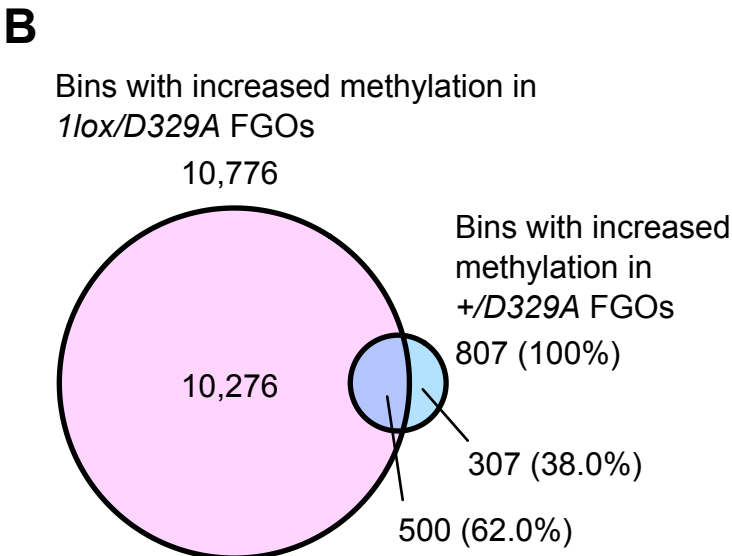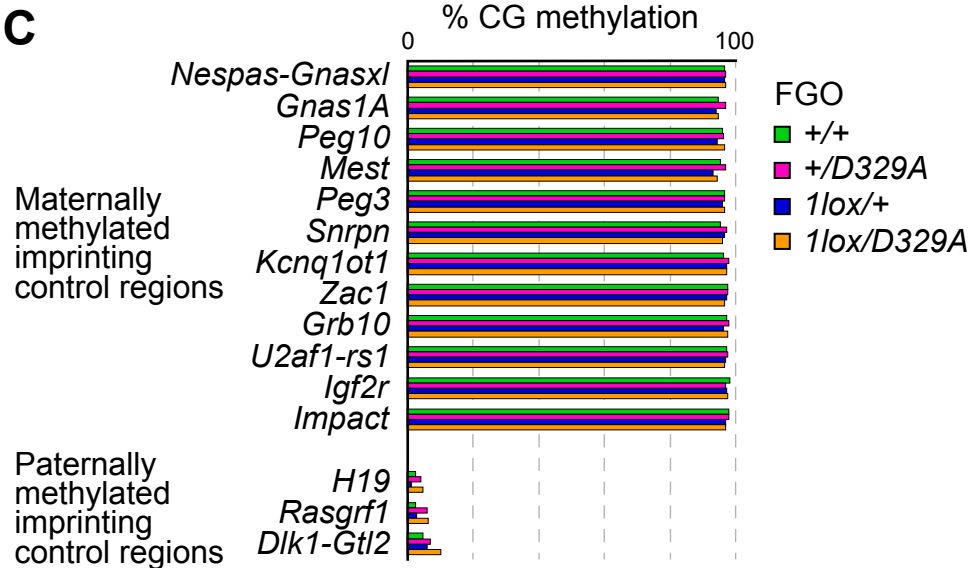

**S3 Fig**

Supplement: S3 Fig — (A) Scatter plots comparing the CG methylation levels of 10-kb bins between wild-type and Dnmt3a1lox/+ FGOs. Bins showing a ≥20% increase and those showing a ≥20% decrease in mutants in comparison to controls are shown by red and blue dots, respectively. (B) A Venn diagram showing the overlap between the hypermethylated bins in Dnmt3a+/D329A and Dnmt3a1lox/D329A FGOs. (C) The CG methylation levels of imprinting control regions in FGOs of the indicated genotypes. (PDF) [file pgen.1009570.s003.pdf]

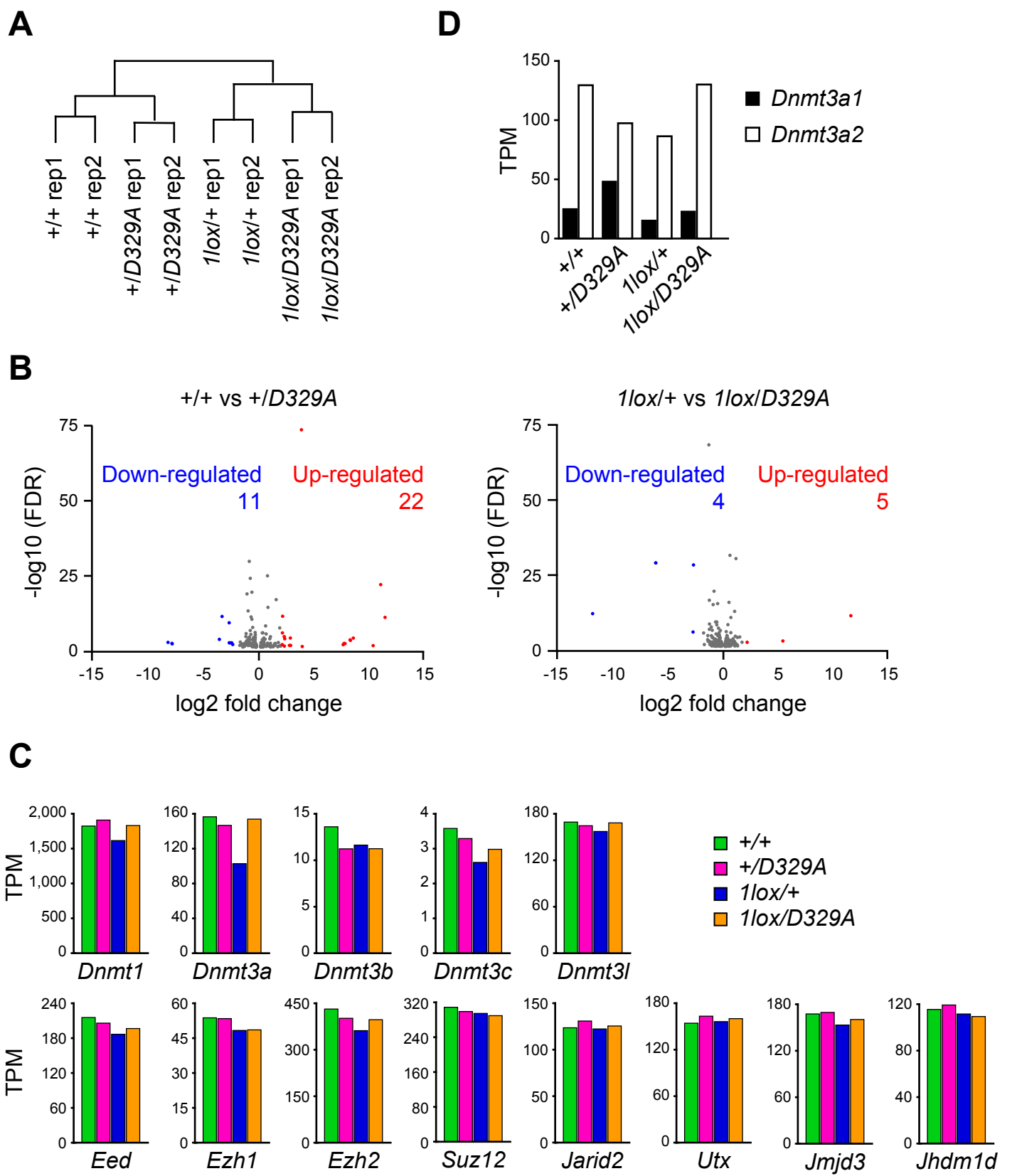

**S4 Fig**

Supplement: S4 Fig — (A) Cluster analysis of the transcriptomes from replicate FGO samples of the indicated genotypes. (B) Volcano plots showing genes differentially expressed between wild-type and Dnmt3a+/D329A FGOs and those between Dnmt3a1lox/+ and Dnmt3a1lox/D329A FGOs. The read counts are the average of the replicates. Blue dots represent down-regulated genes, whereas red dots represent up-regulated genes. (C) Expression of the Dnmt3 family members, Polycomb catalytic core components, and H3K27me3 demethylases in FGOs of the indicated genotypes. The transcript per kilobase million (TPM) values are the average of the replicates. (D) Expression of the Dnmt3a isoforms in FGOs of the indicated genotypes. (PDF) [file pgen.1009570.s004.pdf]

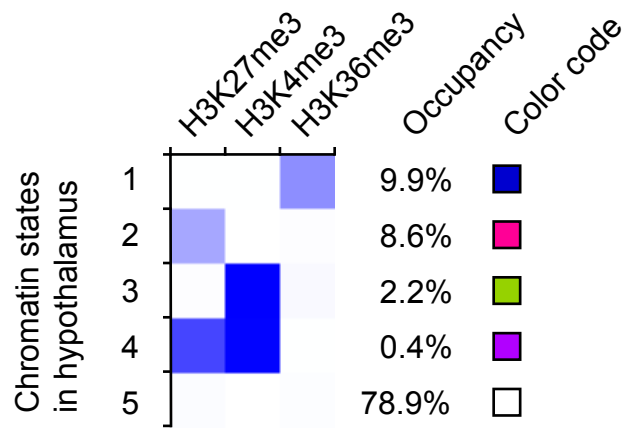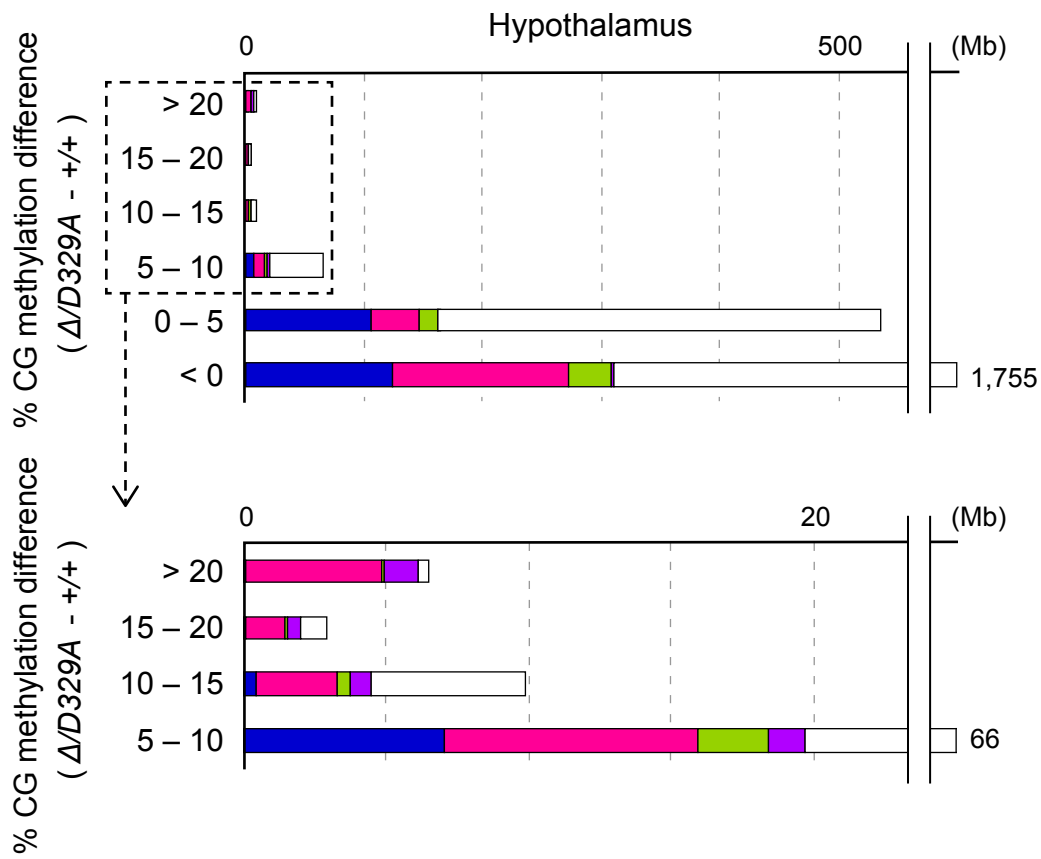

**S5 Fig**

Supplement: S5 Fig — Definition of a five-state model based on three histone H3 marks in the hypothalamus (top). Darker shades of blue in the emission profile represent greater enrichment of the histone marks in each chromatin state. The genomic occupancy of each chromatin state is also shown. Stacked bar charts showing the abundance of each chromatin state in the bulk of 10-kb bins with indicated degrees of CG hypermethylation in the Dnmt3aΔ/D329A hypothalamus in comparison to the wild-type hypothalamus (middle). The bottom panel is a zoom-in view of the middle panel. The chromatin states are indicated by the color code shown at the top. Published histone H3 mark data of the wild-type hypothalamus were used to define the chromatin states [41]. (PDF) [file pgen.1009570.s005.pdf]

**A**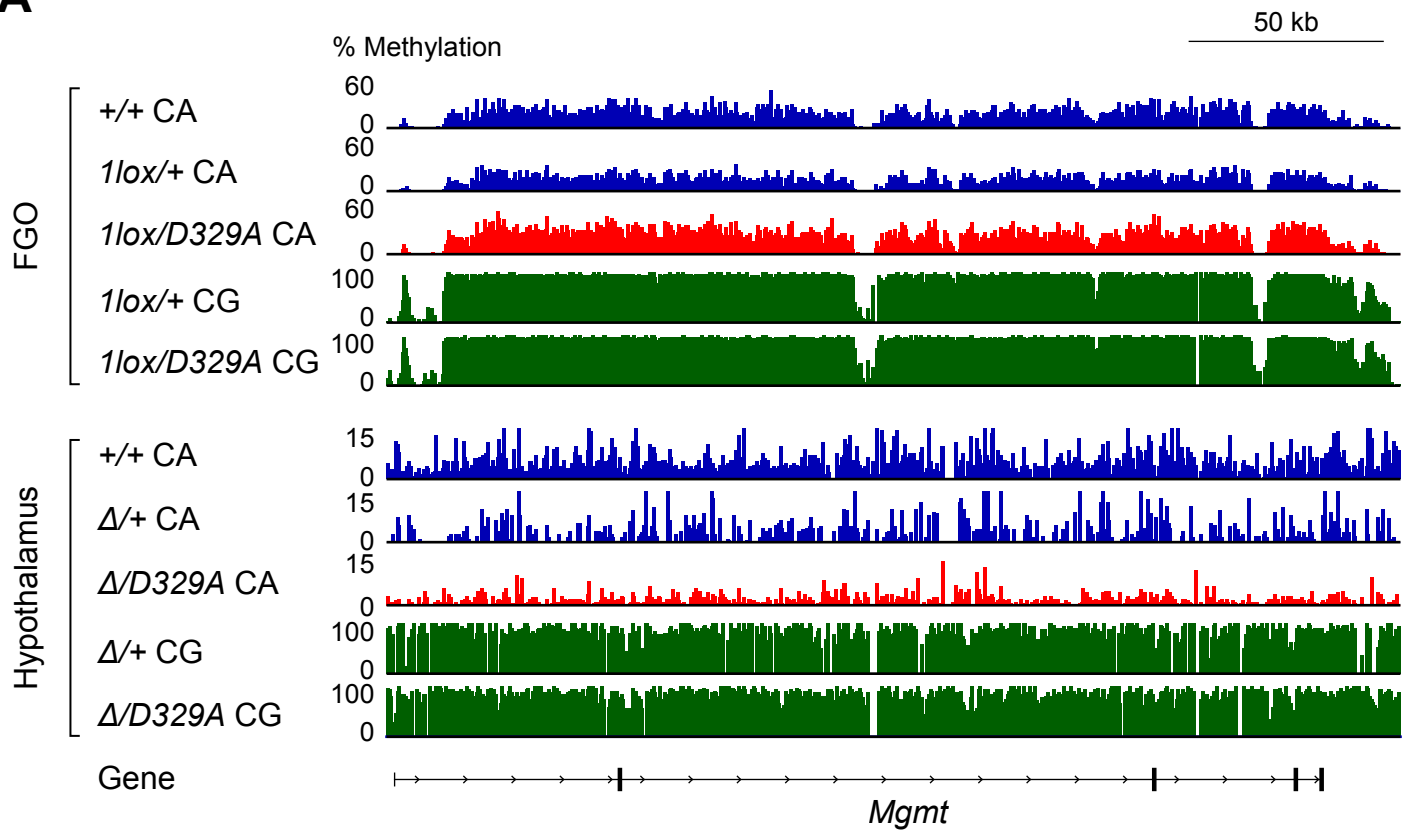**B**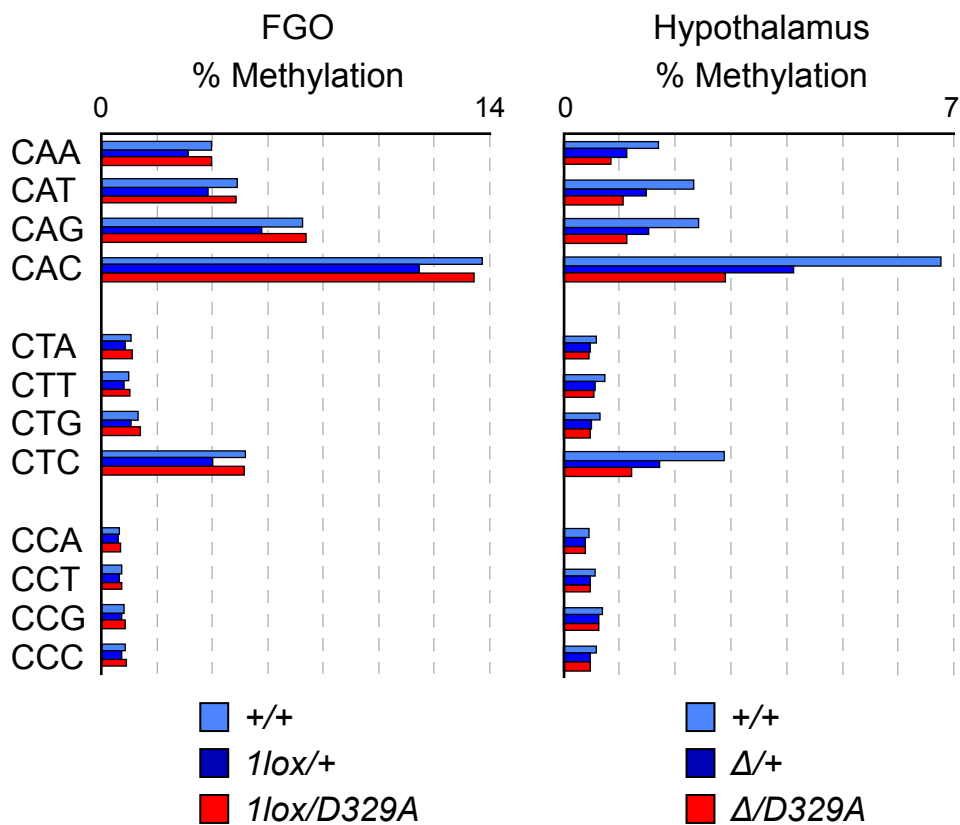**S6 Fig**

Supplement: S6 Fig — (A) A genome browser view showing CA hypermethylation in Dnmt3aD329A oocytes and CA hypomethylation in the Dnmt3aD329A hypothalamus. CG methylation patterns are also shown. (B) Bar graphs showing the methylation levels of the respective CHH trinucleotides in FGOs (left) and the hypothalamus (right) of indicated genotypes. (PDF) [file pgen.1009570.s006.pdf]
