# Supplementary material for: The DNMT3A PWWP domain is essential for the normal DNA methylation landscape in mouse somatic cells and oocytes
Source: PLoS Genet. 2021 May 28;17(5):e1009570. doi: 10.1371/journal.pgen.1009570 (PMC8162659; doi:10.1371/journal.pgen.1009570)
Supplement: S1 Table — (PDF) [file pgen.1009570.s007.pdf]

S1 Table. WGBS and mapping summary

Sample		Sequenced reads	Mapped reads	Average depth	Bisulfite conversion rate	Pearson's correlation coefficient between the replicates*
Tail tip *+/+*	Replicate 1	155,724,488	66,027,515		99.55	
	Replicate 2	194,570,658	88,370,180		99.48	
	Total	350,295,146	154,397,695	6.1		0.91
Tail tip *+/D329A*	Replicate 1	160,306,650	73,483,679		99.55	
	Replicate 2	190,317,336	85,972,328		99.48	
	Total	350,623,986	159,456,007	6.3		0.90
Tail tip *D329A/D329A*	Replicate 1	139,611,314	60,443,737		99.54	
	Replicate 2	167,962,225	71,578,857		99.48	
	Total	307,573,539	132,022,594	5.3		0.89
FGO *+/+*	Replicate 1	505,301,382	201,291,987		99.56	
	Replicate 2	146,686,918	63,184,564		99.49	
	Total	651,988,300	264,476,551	9.1		0.99
FGO *+/D329A*	Replicate 1	493,584,738	218,034,356		99.52	
	Replicate 2	293,042,173	131,010,714		99.49	
	Total	786,626,911	349,045,070	11.9		0.99
FGO *1lox/+*	Replicate 1	736,317,388	339,976,991		99.49	
	Replicate 2	412,936,757	190,930,251		99.53	
	Total	1,149,254,145	530,907,242	18.2		0.99
FGO *1lox/D329A*	Replicate 1	543,847,469	263,348,697		99.47	
	Replicate 2	583,700,308	275,340,566		99.52	
	Total	1,127,547,777	538,689,263	18.4		0.99
FGO *+/+* (P25)		273,128,977	116,715,775	4.0	99.47	
FGO *+/D329A* (P25)		279,921,232	131,125,268	4.6	99.53	
FGO *1lox/+* (P25)		141,804,280	66,794,842	2.3	99.53	
FGO *1lox/D329A* (P25)		135,430,105	62,487,630	2.2	99.55	

* The correlation coefficient of CG methylation levels for the replicates was calculated across all 10-kb bins.
