# Supplementary material for: The DNMT3A PWWP domain is essential for the normal DNA methylation landscape in mouse somatic cells and oocytes
Source: PLoS Genet. 2021 May 28;17(5):e1009570. doi: 10.1371/journal.pgen.1009570 (PMC8162659; doi:10.1371/journal.pgen.1009570)
Supplement: S2 Table — (PDF) [file pgen.1009570.s008.pdf]

S2 Table. RNA-seq and mapping summary

Sample		Sequenced reads	Mapped reads	Pearson's correlation coefficient between the replicates
FGO *+/+*	Replicate 1	33,630,431	30,316,710	
	Replicate 2	49,383,627	44,457,140	
	Total	83,014,058	74,773,850	0.986
FGO *+/D329A*	Replicate 1	48,780,055	43,545,742	
	Replicate 2	42,281,804	37,460,438	
	Total	91,061,859	81,006,180	0.989
FGO *1lox/+*	Replicate 1	53,527,580	46,499,443	
	Replicate 2	32,922,776	28,587,422	
	Total	86,450,356	75,086,864	0.987
FGO *1lox/D329A*	Replicate 1	57,388,928	48,663,969	
	Replicate 2	56,171,688	48,353,385	
	Total	113,560,616	97,017,354	0.989
