# Supplementary material for: The DNMT3A PWWP domain is essential for the normal DNA methylation landscape in mouse somatic cells and oocytes
Source: PLoS Genet. 2021 May 28;17(5):e1009570. doi: 10.1371/journal.pgen.1009570 (PMC8162659; doi:10.1371/journal.pgen.1009570)
Supplement: S3 Table — (PDF) [file pgen.1009570.s009.pdf]

S3 Table. List of differentially expressed genes

Compared genotypes	Regulation	Ensembl ID	Symbol	Log2 fold change	Adjusted P-values
+/+ vs +/D329A	Up	ENSMUSG00000116988	*Gm49673*	11.46	6.59E-12
+/+ vs +/D329A	Up	ENSMUSG00000098950	*Gm28036*	11.07	8.30E-23
+/+ vs +/D329A	Up	ENSMUSG00000094365	*Gm21982*	10.38	1.69E-02
+/+ vs +/D329A	Up	ENSMUSG00000042155	*Klhl23*	8.59	5.19E-05
+/+ vs +/D329A	Up	ENSMUSG00000113816	*Gm47003*	8.33	3.03E-04
+/+ vs +/D329A	Up	ENSMUSG00000102046	*2810404M03Rik*	8.30	2.02E-04
+/+ vs +/D329A	Up	ENSMUSG00000020674	*Pxdn*	8.29	0.00E+00
+/+ vs +/D329A	Up	ENSMUSG00000079444	*Gm21981*	7.84	3.57E-03
+/+ vs +/D329A	Up	ENSMUSG00000103255	*Pcdhac1*	7.75	3.10E-03
+/+ vs +/D329A	Up	ENSMUSG00000037390	*Muc3*	7.67	8.19E-03
+/+ vs +/D329A	Up	ENSMUSG00000091572	*Vmn2r3*	3.96	3.33E-02
+/+ vs +/D329A	Up	ENSMUSG00000107092	*Gm7993*	3.89	2.35E-74
+/+ vs +/D329A	Up	ENSMUSG00000081800	*Gm14200*	2.91	1.46E-02
+/+ vs +/D329A	Up	ENSMUSG00000037035	*Inhbb*	2.88	5.01E-05
+/+ vs +/D329A	Up	ENSMUSG00000091613	*Gm17046*	2.80	1.42E-02
+/+ vs +/D329A	Up	ENSMUSG00000038855	*Itpkb*	2.38	8.77E-05
+/+ vs +/D329A	Up	ENSMUSG00000050248	*Evc2*	2.34	2.51E-02
+/+ vs +/D329A	Up	ENSMUSG00000037071	*Scd1*	2.33	1.68E-05
+/+ vs +/D329A	Up	ENSMUSG00000117286	*Gm1043*	2.19	2.81E-12
+/+ vs +/D329A	Up	ENSMUSG00000117286	*Jakmip1*	2.19	2.81E-12
+/+ vs +/D329A	Up	ENSMUSG00000050953	*Gja1*	2.17	9.79E-07
+/+ vs +/D329A	Up	ENSMUSG00000027840	*Wnt2b*	2.15	7.67E-03
+/+ vs +/D329A	Down	ENSMUSG00000105340	*Gm42878*	-25.80	3.04E-05
+/+ vs +/D329A	Down	ENSMUSG00000116024	*Gm49527*	-8.20	1.39E-03
+/+ vs +/D329A	Down	ENSMUSG00000032083	*Apoa1*	-7.84	3.07E-03
+/+ vs +/D329A	Down	ENSMUSG00000116798	*Gm49591*	-7.82	4.32E-03
+/+ vs +/D329A	Down	ENSMUSG00000074934	*Grem1*	-3.54	1.41E-04
+/+ vs +/D329A	Down	ENSMUSG00000080268	*Brms1*	-3.30	3.34E-12
+/+ vs +/D329A	Down	ENSMUSG00000031302	*Nlgn3*	-2.66	4.18E-10
+/+ vs +/D329A	Down	ENSMUSG00000060404	*Olfr1369-ps1*	-2.65	1.94E-03
+/+ vs +/D329A	Down	ENSMUSG00000116358	*Gm49450*	-2.57	2.06E-03
+/+ vs +/D329A	Down	ENSMUSG00000063954	*Hist2h2aa2*	-2.43	1.98E-03
+/+ vs +/D329A	Down	ENSMUSG00000089945	*Pakap*	-2.33	7.21E-03
1lox/+ vs 1lox/D329A	Up	ENSMUSG00000107877	*Gm43951*	23.27	2.58E-04
1lox/+ vs 1lox/D329A	Up	ENSMUSG00000096056	*Gm21986*	11.54	3.35E-12
1lox/+ vs 1lox/D329A	Up	ENSMUSG00000020674	*Pxdn*	8.55	1.46E-298
1lox/+ vs 1lox/D329A	Up	ENSMUSG00000000183	*Fgf6*	5.38	8.60E-04
1lox/+ vs 1lox/D329A	Up	ENSMUSG00000113428	*Gm7664*	2.14	2.34E-03
1lox/+ vs 1lox/D329A	Down	ENSMUSG00000073197	*5730507C01Rik*	-11.85	6.78E-13
1lox/+ vs 1lox/D329A	Down	ENSMUSG00000116744	*Gm49764*	-6.13	8.87E-30
1lox/+ vs 1lox/D329A	Down	ENSMUSG00000098374	*Gm28043*	-2.76	9.05E-07
1lox/+ vs 1lox/D329A	Down	ENSMUSG00000031302	*Nlgn3*	-2.71	4.21E-29
