# Supplementary material for: The DNMT3A PWWP domain is essential for the normal DNA methylation landscape in mouse somatic cells and oocytes
Source: PLoS Genet. 2021 May 28;17(5):e1009570. doi: 10.1371/journal.pgen.1009570 (PMC8162659; doi:10.1371/journal.pgen.1009570)
Supplement: S4 Table — (PDF) [file pgen.1009570.s010.pdf]

S4 Table. Oligonucleotide sequences

	Sequence (5' to 3')
(Guide RNA)	
Dnmt3a_3896094-3896113_Fwd	CACCAGCAGCTGAAGGCACTCGCT
Dnmt3a_3896094-3896113_Rev	AAACAGCGAGTGCCTTCAGCTGCT
(Single-stranded donor oligodeoxynucleotide)	
ssODN_Dnmt3a_PWWP	GGCCGGAGCCGAGCAGCTGAAGGCACTCGCTGGGTCATGTGGTTCGGAGCTGGCAAGTTCTCAGTGGTGAGTTGGACTTGGTG
(Genotyping primers)	
Dnmt3a^{D329A}_Eprobe	GGTZ[1]CGGAGCTGG
Dnmt3a^{D329A}_Fwd	AGGCCGAATTGTGTCTTGGT
Dnmt3a^{D329A}_Rev	TGATGGTGGCTATGGCTTCTTC
Zp3-Cre_Fwd	GCAGAACCTGAAGATGTTCGCGAT
Zp3-Cre_Rev	AGGTATCTCTGACCAGAGTCATCC
Dnmt3a_2lox_Fwd	CTGTGGCATCTCAGGGTGATGAGCA
Dnmt3a_2lox_Rev	GCAAACAGACCCAACATGGAACCCT

[1]Z: Thiazole orange-labeled T
